# Comparison of ground reaction force measurements in a population of Domestic Shorthair and Maine Coon cats

**DOI:** 10.1371/journal.pone.0208085

**Published:** 2018-12-12

**Authors:** Eva Schnabl-Feichter, Alexander Tichy, Michaela Gumpenberger, Barbara Bockstahler

**Affiliations:** 1 Department for Companion Animals and Horses, University Clinic for Small Animals, Small Animal Surgery, University of Veterinary Medicine, Veterinärplatz, Vienna, Austria; 2 Department for Biomedical Science, Platform Bioinformatics and Biostatistics, University of Veterinary Medicine, Veterinärplatz, Vienna, Austria; 3 Department for Companion Animals and Horses, University Clinic for Radiology, University of Veterinary Medicine, Veterinärplatz, Vienna, Austria; 4 Department for Companion Animals and Horses, University Clinic for Small Animals, Small Animal Surgery, Section for Physical Therapy, University of Veterinary Medicine, Veterinärplatz, Vienna, Austria; Faculty of Animal Sciences and Food Engineering, University of São Paulo, BRAZIL

## Abstract

Current research on gait analysis mostly involves horses and dogs. Feline kinetics and kinematics are being investigated and receiving more clinical interest at present. Ground reaction forces measured on pressure-sensitive mattresses have been established in healthy Domestic Shorthair cats (DSH). Currently, no further information exists on either breed-specific measured gait reaction forces or comparisons among breeds. Because Maine Coon (MC) cats appear to be over-represented with orthopaedic diseases of the hind limb (hip dysplasia, patellar luxation), we evaluated ground reaction force GRF measurements in MC cats and compared them with those of DSH cats. Pre-evaluation radiological and clinical exams determined that the cats were not lame. The parameters evaluated were peak vertical force (PFz), vertical impulse (IFz), time to PFz (TPFz), step length (SL), paw contact area (PCA), stance phase duration (SPD) and symmetry index (SI) for the fore- and hind limbs. In both breeds, PFz and IFz were greater in forelimbs than in hind limbs. The PFz and IFz in Newtons were higher in the MC cats compared to the DSH cats, but not after normalisation for total force (%TF) and body mass (%BM). Furthermore, due to their body conformation, MC cats have a longer SL, larger PCA, and higher body weight than DSH cats. No other parameters differed significantly, except that the TPFz displayed an earlier value in the MC hind limbs. Measured symmetry indices were similar to those reported in dogs and did not differ between breeds. This is the first study to report GRF values and temporospatial parameters in a healthy MC cat population. However, our results could not confirm differences between normalized PFz and IFz and temporospatial parameters between the breeds. The authors therefore conclude that genetic or other causes may be involved in orthopaedic hind limb pathogenesis seen in MC cats more often than in other breeds.

## Introduction

Compared with other species (canines and equines), little has been published on feline kinetics and kinematic biomechanics [1, 2]. Recently, more studies have been conducted to investigate normal and abnormal gait in cats [2–10], as cats are receiving more attention than as orthopaedic patients, and pressure-sensitive walkways (PSWs) make these measurements easier. PSWs allow cats to walk at their own pace without being restrained. Many pressure sensors are present with PSWs; these sensors facilitate quantifying both high and low pressure responses [11, 12]. Parameters most commonly evaluated include orthogonal GRFs (Peak Vertical Force and Vertical Impulse, a function of force and time), that result from contact between the paw and the ground during gait [1, 13]. Additional parameters that we evaluated include loading rate, temporal gait characteristics, and paw pressure distribution [8].

Results from available studies have shown that healthy cats exert greater force in their forelimbs than their hind limbs. Forelimb PFz ranges from 48.2% to 62.0% of body mass (BM) [3–7, 10], and hind limb PFz ranges from 38.3% to 50.2% of BM [3, 5, 7, 10]. Forelimb IFz ranges from 12.7% to 19.4% of BM [3–7, 10] and hind limb IFz ranges from 13.1% to 14.6% of BM [3, 5, 7, 10]. These findings show that cats, like other quadrupeds, develop propulsion with their hind limbs, while the main weight bearing occurs on their front limbs. Investigations of force distribution in the paws further demonstrated that the mean weight during a strike is transferred from the caudal to the craniomedial portion of the paw [2].

No reports to date have considered interbreed differences in feline kinetic gait metrics. In domestic dogs, investigators have compared GRF among dogs and cats [8], dog breeds [14–16], and sizes [17]. Although cats have similar body confirmations, some purebred cats present with orthopaedic diseases more frequently than others [18–21]. One example is Maine Coon cats (MC), over-represented in diagnoses of slipped capital physis and coxarthrosis [22, 23]; while the cause(s) of this over-representation remain unclear.

We designed this study using client-owned cats and PSWs to obtain GRF data from healthy purebred MC cats, compared with DSH cats. We hypothesized that MC cats exert greater forces in their hind limbs than DSH, and that this may be one explanation for MC cats being more prone to hind limb orthopaedic diseases. We further hypothesized that MC cats will have a longer stride length and larger paw contact area than DSH cats.

## Materials and methods

### Cats

This study was discussed and approved by the Institutional Ethics and Animal Welfare Committee of the veterinary university vienna in accordance with Good Scientific Practice guidelines and national legislation (reference number 13/10/2017).

We recruited fifteen client-owned DSH cats, evaluated during a previous study [10], and fifteen client-owned MC cats. All owners signed a written informed consent. A board-certified surgeon (ESF) performed complete orthopaedic and clinical examination of all cats. Additionally, radiographs of the hips, stifles, and elbows were made, following standard procedural protocols. Signs of orthopaedic disease, including lameness, pain, joint swelling, reduced range of motion, abnormal clinical exam, or relevant pathological changes in any joint, precluded cats from participating in the study.

Among the 15 DSH cats that met the criteria for study inclusion, 7 were neutered males and 8 were spayed females, with mean body mass (± SD) of 5.5 ± 1.2 kg (range: 4.0–6.6 kg) and mean age of 7.2 ± 4.2 years (range: 2.6–14.9 years). The 15 MCs included 7 intact males, 3 neutered males, 4 intact females and one spayed female. The mean body mass (± SD) was 6.3 ± 1.5 kg (range: 3.1–8.5 kg), and the mean age was 3.7 ± 2.7 years (range: 0.5–9.1 years).

### Experimental set-up and equipment

All clinical and radiographic examinations and measurements were conducted at the University of Veterinary Medicine, Vienna, Austria. Gait was analysed in a quiet room with the owner and two researchers present. A Zebris FDM Type 2 pressure plate (Zebris Medical GmbH, Allgäu, Germany) was mounted in the middle of a 7-m runway. The pressure plate was 203.2 x 54.2 cm in size and included 15,360 sensors with a sampling rate of 100 Hz. The pressure sensitive plate was covered with a rubber mat to prevent slipping and to be hidden from the cats.

After the clinical and orthopaedic exam, each cat was allowed a few minutes of free movement in the gait analysis room to facilitate acclimation. After this period, the cat was stimulated with toys, food, and verbal and visual stimuli, to cross the pressure-sensitive mat in a straight line. In some instances, a portable cartoon wall was placed alongside the plate to keep the cat moving in the same direction. Each cat could determine its own walking pace over the pressure plate. The measurement was defined as valid if the cat crossed the plate at least three times, allowing at least five valid step cycles to be measured. Any change in velocity (trot or stop) or turning of the head during the gait cycle was an exclusion criterion.

All measurements were video-recorded using a Panasonic NV-MX500 camera, and data were stored using WinFDM software (v1.2.2; Zebris Medical GmbH) and processed using specially-developed software (Pressure Analyzer 1.3.0.2; Michael Schwanda).

The study was executed as a non-randomised prospective trial, where the DSH group data were taken from a previously published study [10]. From the previous study, the authors chose the trials with the velocity that was comparable to the MC cats (range: 0.52 m/s to 0.83 m/s for DSH cats, 0.52 m/s and 1.1 m/s for MC cats).

### Data processing and outcome parameters

Gait velocity (V) was recorded from the left forelimb. Gait parameters evaluated were PFz (N); IFz (N); time to PFz (TPFz, %StPh); step length (SL; m); paw contact area (PCA; cm^2^); and stance phase duration (SPD; s). The GRF data, initially displayed in Newtons (N), were normalized to body mass (%BM) and total force (%TF). A symmetry index (SI%) of the forelimbs and hind limbs was calculated from the PFz and IFz.

The SI of the contralateral limb pair was calculated using the formula modified from Budsbergh et al [10, 24].

$$SIXFz=abs\left(\frac{\left(XFzFL-XFzFR\right)}{\left(XFzFL+\;XFzFR\right)}\right)\times 100$$

where the SI = symmetry index, X = the given value of PFz or IFz, abs = absolut, FL = front left, and FR = front right. Symmetry was calculated so that an SI of 0% would represent perfect paired-limb symmetry.

### Statistical methods

All evaluated data were processed with IBM SPSS statistical software, version 24. The Kolmogorov-Smirnov test was used to determine normal distribution. Descriptive statistics were calculated for each parameter and breed. A general linear model (GLM) was used to compare data of the left and right forelimbs and hind limbs, and between breeds. Each parameter was compared using an independent sample t-test to detect statistically significant differences between the DSH and MC cats. Data are presented as the mean and SD. A *p*-value < 0.05 was considered statistically significant.

## Results

All data were normally distributed. Mean values of all parameter measurements are shown in Table 1. DSH body mass ranged from 3.8 kg to 6.6 kg (mean 4.97 ± 1.11 kg), while MC body mass ranged from 3.1 kg to 8.5 kg (mean 6.25 ± 1.53 kg). MC cats yielded significantly higher body mass (p = 0.01).

**Table 1:** Descriptive statistics of 15 tested DSH and 15 Maine Coons and all legs and comparison of their ground reaction forces and temporospatial parameters.

Limb, laterality	DSH Mean	± SD	Range	MC Mean	± SD	Range	Limb, laterality	DSH Mean	± SD	Range	MC Mean	± SD	Range
**Fore, left**							**Fore, right**						
PFz, N	27.39 ^a^^,^^b^	6.58	19.73–39.64	35.59^a^^,^^b^	10.89	16.44–54.43	PFz, N	27.47 ^a^^,^^b^	6.43	20.25–39.46	35.85 ^a^^,^^b^	11.06	16.77–56.14
PFz, % BM	56.23 ^b^	4.71	50.19–65.17	57.22 ^b^	5.68	47.82–67.13	PFz, % BM	56.51 ^b^	5.10	48.08–65.26	57.70 ^b^	5.80	48.74–70.39
PFz, % TF	28.98 ^b^	2.33	26.13–35.54	28.47 ^b^	2.06	25.41–32.12	PFz, % TF	29.12 ^b^	2.55	25.37–34.82	28.71 ^b^	2.10	25.83–33.43
TPFz, % StPh	58.80 ^b^	5.65	48.25–68.59	58.04 ^b^	7.38	44.17–68.31	TPFz, % StPh	58.36 ^b^	7.35	45.99–67.24	58.15 ^b^	7.86	39.79–70.12
IFz, N	9.02 ^a^^,^^b^	3.08	5.70–15.73	12.92 ^a^^,^^b^	4.80	4.30–21.03	IFz, N	9.02 ^a^^,^^b^	3.20	5.75–15.63	13.02 ^a^^,^^b^	4.88	4.71–23.24
IFz, % BM	18.30 ^b^	3.56	12.92–25.87	20.40 ^b^	3.91	11.25–27.63	IFz, % BM	18.28 ^b^	3.63	13.03–25.70	20.58 ^b^	3.72	12.30–27.87
IFz, % TF	29.29 ^b^	2.42	25.75–34.61	29.28 ^b^	2.68	24.83–33.89	IFz, % TF	29.23 ^b^	2.53	26.05–35.20	29.61 ^b^	2.41	25.47–33.29
SPD, s	0.45 ^b^	0.06	0.34–0.56	0.51 ^b^	0.09	0.33–0.69	SPD, s	0.45 ^b^	0.07	0.35–0.57	0.51 ^b^	0.10	0.35–0.75
SL, m	0.49 ^a^	0.04	0.41–0.56	0.56 ^a^	0.08	0.41–0.68	SL, m	0.50 ^a^	0.04	0.41–0.56	0.57 ^a^	0.07	0.47–0.70
PCA, cm^2^	12.79 ^a^	1.59	10.40–15.78	15.22 ^a^	2.28	10.96–18.29	PCA, cm^2^	12.58 ^a^	1.35	10.42–15.24	15.37 ^a^	2.55	10.94–20.09
**Hind, left**							**Hind, right**						
PFz, N	20.20 ^a^^,^^b^	6.03	8.88–30.86	26.26 ^a^^,^^b^	6.75	13.86–41.56	PFz, N	19.92 ^a^^,^^b^	5.74	10.04–29.80	26.24 ^a^^,^^b^	7.43	13.64–43.58
PFz, % BM	41.19 ^b^	7.08	23.82–50.28	43.03 ^b^	3.92	34.76–49.85	PFz, % BM	40.72 ^b^	6.82	26.94–52.79	42.89 ^b^	5.12	32.68–52.27
PFz, % TF	21.07 ^b^	2.67	13.91–24.22	21.45 ^b^	1.81	18.13–24.85	PFz, % TF	20.83 ^b^	2.32	15.73–24.49	21.37 ^b^	2.32	16.57–24.33
TPFz, % StPh	45.17 ^a^^,^^b^	9.51	34.96–60.94	38.71 ^a^^,^^b^	6.20	30.04–49.31	TPFz, % StPh	47.91 ^b^	10.43	27.76–67.77	41.64 ^b^	6.52	32.01–60.91
IFz, N	6.54 ^a^^,^^b^	2.50	2.50–11.02	8.88 ^a^^,^^b^	3.19	3.81–15.37	IFz, N	6.43 ^a^^,^^b^	2.42	2.56–11.19	8.88 ^a^^,^^b^	3.39	3.95–16.74
IFz, % BM	13.20 ^b^	3.34	6.71–18.97	14.33 ^b^	3.10	8.69–21.07	IFz, % BM	13.03	3.33	6.86–18.92	14.32	3.34	9.16–20.21
IFz, % TF	20.88 ^b^	2.49	14.93–24.06	20.57 ^b^	2.35	15.98–24.14	IFz, %TF	20.60	2.45	15.26–24.06	20.53	2.66	16.29–24.40
SPD, s	0.43 ^b^	0.06	0.34–0.53	0.48 ^b^	0.11	0.33–0.76	SPD, s	0.43 ^b^	0.06	0.33–0.54	0.48 ^b^	0.09	0.34–0.70
SL, m	0.51 ^a^	0.06	0.42–0.61	0.57 ^a^	0.08	0.49–0.70	SL, m	0.51 ^a^	0.05	0.43–0.60	0.58 ^a^	0.08	0.47–0.69
PCA, cm^2^	12.30 ^a^	2.25	8.53–16.14	15.15 ^a^	2.30	10.35–20.38	PCA, cm^2^	12.27 ^a^	2.27	9.33–16.37	14.90 ^a^	2.03	10.33–18.91

DSH = Domestic Short Hair, MC = Maine Coon, Mean = Mean of the three measurements, SD = Standard deviation, N = Newton, BM = Body mass, TF = Total force, StPh = Stance Phase, PFz = Peak vertical force, IFz = Vertical impulse, TPFz = Time to PFz, SPD = Stance phase duration, SL = Step length, PCA = Paw contact area.

^a^ significant difference between breeds (p < 0.05),

^b^ significant difference between fore- and hind limb and leg (p < 0.05)

Gait velocity ranged from 0.52 m/s to 0.83 m/s (mean 0.7 ± 0.09 m/s) in DSH cats, while MC cats’ gait velocity ranged from 0.52 m/s to 1.1 m/s (mean 0.74 ± 0.17 m/s). Mean gait velocity was not different between breeds (p> 0.05).

### Ground reaction forces (PFz, IFz)

The PFz and IFz significantly differed between the groups, where MC cats displayed a higher PFz (p = 0.01) and IFz (p = 0.02) in Newtons, but not after normalising for %BM and %TF. The breeds did not differ between the left and right limbs for PFz or IFz in Newtons or after normalisation. However, both groups significantly differed between the fore- and hind limbs (p< 0.01) in N and after normalisation (% BM, % TF), with the forelimbs displaying higher values (Table 1).

### Symmetry index (SI)

The SI in the forelimb PFz was 1.35 ± 0.89% (0.01–3.39) in DSH and 1.53 ± 0.84% (0.50–3.53) in MC cats. In the hind limbs, it was 3.14 ± 1.79% (0.72–6.38) in DSH and 2.02 ± 1.25% (0.25–4.50) in MC cats. In contrast, the SI in the forelimb IFz was 1.86 ± 1.39% (0.15–4.76) in DSH and 2.44 ± 2.01% (0.22–5.37) in MC cats. In the hind limbs, it was 2.40 ± 2.34% in DSH (0.01–6.89) and 3.17 ± 1.79% (0.56–7.11) in MC cats.

The GLM revealed a significant difference in the SI PFz (%) between the hind and forelimbs (p< 0.01) and a significant influence by breed (p< 0.05), as the SI PFz (%) in the DSH hind limbs was significantly higher than their forelimbs (p< 0.01).

### Paw contact area (PCA)

**Fig 1:**
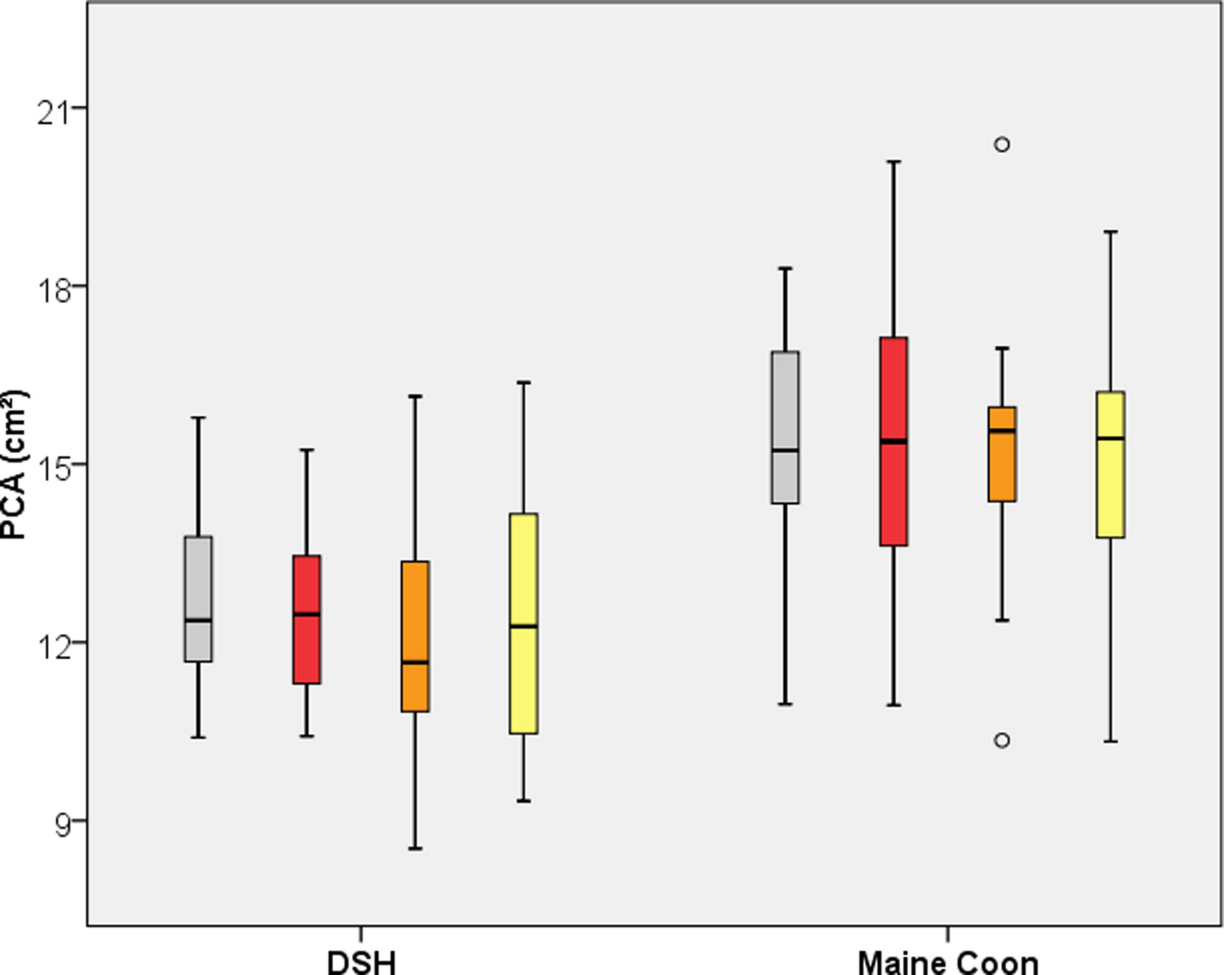
Boxplot of the Paw Contact Area (PCA) for Domestic Shorthair and Maine Coon Cats. Grey = left forelimb; red = right forelimb; orange = left hind limb; yellow = right hind limb.

Table 1 and Fig 1 display the PCA of both breeds for all legs. The PCA of the MC was significantly larger (p< 0.01) than in the DSH cats, with a mean value of 12.49 ± 1.87 cm^2^ for all four paws in the DSH population and of 15.16 ± 2.29 cm^2^ for the MC cats. Right and left legs and fore and hind limbs did not differ.

### Step length (SL)

**Fig 2:**
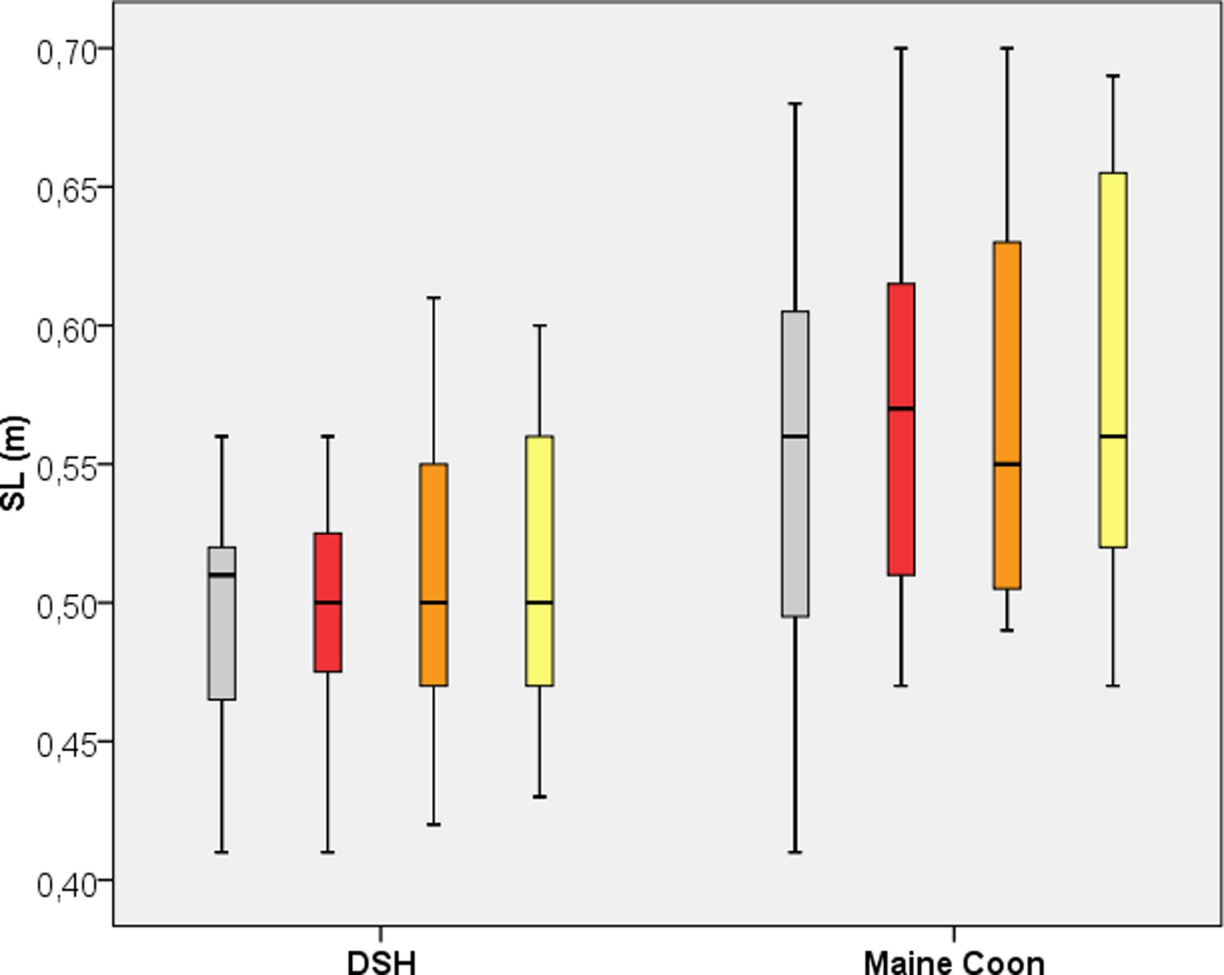
Boxplot of the Step Length (SL) for Domestic Shorthair and Maine Coon Cats. Grey = left forelimb; red = right forelimb; orange = left hind limb; yellow = right hind limb.

Table 1 and Fig 2 display the SL for both breeds and all legs, where the SL was 0.50 ± 0.05 m in DSHs and 0.57 ± 0.07 m in MCs. The MC SL was significantly longer (p< 0.01) than the DSH SL.

### Stance phase duration (SPD)

**Fig 3:**
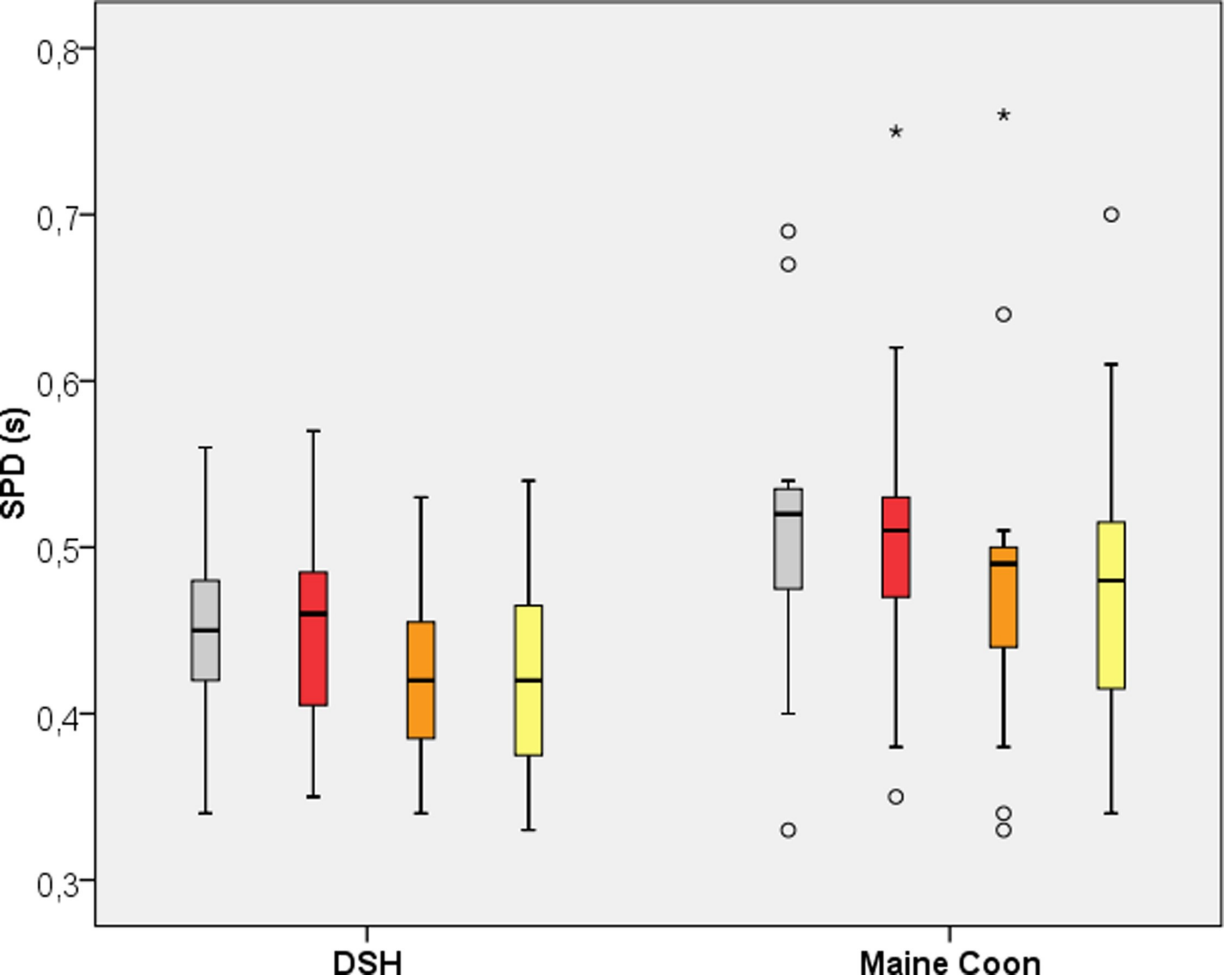
Boxplot of the Stance Phase Duration (SPD) for Domestic Shorthair and Maine Coon Cats. Grey = left forelimb; red = right forelimb; orange = left hind limb; yellow = right hind limb.

Table 1 and Fig 3 display the SPD for both breeds and all legs. The fore- and hind limbs significantly differed in each breed (p< 0.01), with the forelimbs having a longer stance phase than the hind limbs.

### Time to PFz (%StPh)

**Fig 4:**
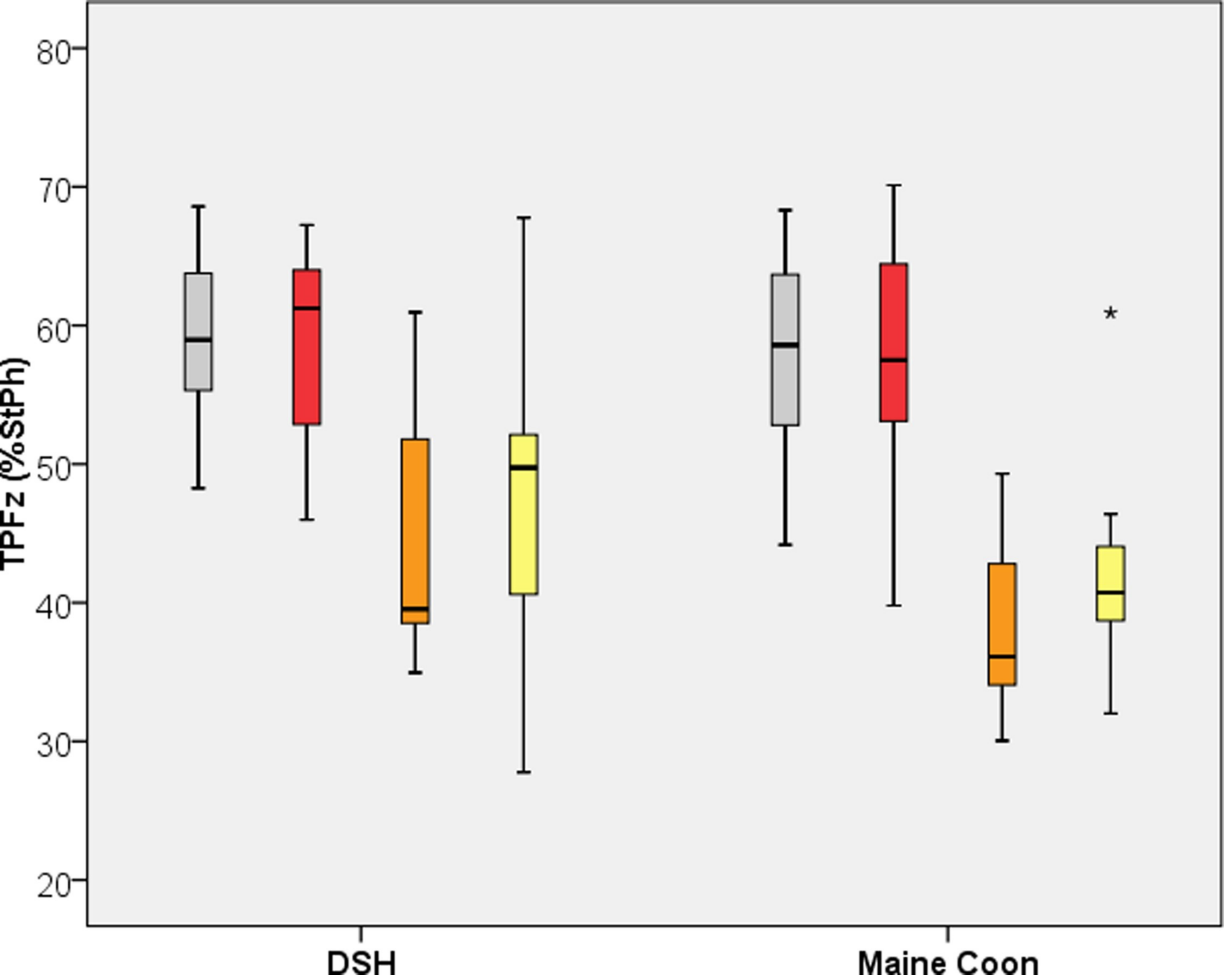
Boxplot of the Time to PFz (TPFZ) for Domestic Shorthair and Maine Coon Cats. Grey = left forelimb, red = right forelimb, orange = left hind limb, yellow = right hind limb.

Table 1 and Fig 4 display the TPFz (%StPh) for both breeds and all legs. Both breeds differed significantly in their fore- and hind limbs, with a later TPFz in the forelimbs (p< 0.01). The forelimb TPFz was not different between the two breeds, but there was a difference between the breeds in the hind limbs. In MC cats displayed an earlier TPFz (p = 0.04) in the left hind limb compared to DSH cats, whereas the right hind limb results did not differ (p = 0.07) between breeds.

## Discussion

Our study provided GRF and temporospatial data for a group of healthy purebred MC cats. Moreover, we compared their results with a group of DSH cats from a previous study [10]. With the available data, we could not confirm the first part of our hypothesis, that MC cats produce greater gait forces in their hind limbs than DSH cats, when GRFs are normalized for BM or TF. However, we did confirm that MC cats have a longer stride length, a larger paw contact area, and a higher body weight than DSHs in this study.

Differences in GRF within a study group can be due to different velocities during measuring [14, 15]. Because data evaluation on a pressure-sensitive walkway is more difficult to obtain in cats compared to leash-walked dogs [5, 7], investigators must be certain that the subjects’ gait velocities are similar; excessively rapid gait trials likely need to be repeated. To avoid this problem, we chose the gait cycle in a previously measured DSH population, which yielded similar velocities. Consequently, our gait velocity ranged from 0.5 m/s to 1.1 m/s, which is comparable with that of other studies [5, 7, 9, 10]. Although the mean measured velocity in MC cats was slightly higher (0.74 m/s vs 0.70 m/s), the two groups did not differ statistically. Thus, we were able to compare our data between the two breeds. Despite the fact that we did not find a difference in GRF between the two groups, further rearch should investigate more deeply the influence of different gait velocities on GRF in this species.

The longer stride length and larger paw contact area measured in the MC cats might result from their larger body size. This finding also is reported from canine studies, when different breeds have been compared. Interestingly, even if one breed had a longer stride length, gait velocity did not differ between breeds in one study [17]. The slightly higher velocity in MC cats could be related to a longer stride. The highest velocity (1.1 m/s) was measured in a MC cat.

The GRF results ranged similarly to those reported earlier [1]. Most feline GRFs reported to date were collected from DSH cats [1, 2]. Among domestic dogs, different breeds have been measured to evaluate the influence of body mass and conformation on gait [14, 15, 17]. In a study by Bertram et al. [14], the investigators found no major gait differences between Labrador Retrievers and Greyhounds during trotting. However, there were some subtle changes that included: (a) the proportion of the stride in which the forefoot was in contact with the ground, (b) the timing of initial hind foot contact relative to initial forefoot contact; and (c) the vertical force distribution between the fore- and hind limbs. The authors concluded that these small differences were due mainly to differences in animal size. Another study comparing Rottweilers and Labrador Retrievers showed that peak vertical forces in the Rottweilers’ thoracic limbs were significantly lower, and vertical impulses in their thoracic and pelvic limbs were significantly higher than in Labradors. However, after the investigators removed the effect of body weight with normalization, no significant differences existed between breeds [15]. Kim et al. [17] compared small and large dogs with the same result as other authors. Mean values of non-normalized GRFs for forelimbs and hind limbs were significantly smaller in small dogs than in large dogs, but when normalized for BM, no significant differences were noted.

We report the first kinetic data measured by a pressure-sensitive walkway in a population of healthy, client-owned MC cats. Similar to previous reports in dogs [15, 17], GRFs in Newtons (non-normalized) were larger in MC cats due to their greater body weight. This is explained with Newton´s second law, where force is a function of mass. However, when forces in our study were normalized to %BM or %TF, this effect was no longer significant. In general, GRF´s data are normalized to %BM [2, 5]. Voss et al. [25] were the first to describe normalization to BM and body size instead, especially for time-associated variables like IFz and SPD. In our previous study, we showed that the coefficient of variation of IFz became more reliable when data are normalized to %TF [10]. Our current data is displayed in N and after normalisation to %BM and %TF, yet it might be speculated that body sizes in cats overall are not that different. However a limitation of our study is, that we have not measured limb length and estimated body size. Therefore, it would be a possibility to include morphometric parameters like body height and length to get more information and a better understanding of breed differences.

In summary, we cannot conclude that MC cats generate greater forces in their fore- or hind limbs than DSH cats. As a result, greater force generation in the hind limb is unlikely to be a part of the pathogenesis of the major orthopaedic disorders seen more often in MCs than in other domestic cat breeds [22, 23].

MC cats develop hip dysplasia more often, possibly due to their narrower gene pool or an unknown inheritance pattern, rather than a different force generation [19, 20]. This also is supported by Polzin’s study [23], in which no correlation was found between a smaller Norberg Angle and developing osteoarthrosis at a higher body weight. The higher incidence (12-fold) of slipped capital femoral epiphysis is unlikely to be driven by greater force in the hind limbs and greater body weight than other breeds. At least in part, the epiphyseal observations could be due to early neutering (where that occurs) and delayed physeal closure (mean age 19 months) in some instances, as previously discussed by other authors [22].

It is our view that the most interesting finding between DSH and MC cats was the time to PFz. An earlier time to maximal PFz (%StPH) was seen in both hind limbs for MC cats (left hind limb statistically significant). Our assessment is that this is not due to a larger step length with hind limbs because step length was not different between fore- and hind limbs of both cat breeds. The difference also was not the result of higher GRF between the breeds or a longer stance phase duration, as we found no statistical significance when comparing the data. One reason for the earlier PFz (%StPh) may be a different joint angulation in MC than in the smaller DSH. Whether this difference is part of the pathogenesis of a greater prevalence of some orthopaedic disorders is unknown to date.

Gait symmetry evaluations often are used by researchers as tools to detect gait dysfunction and as diagnostic tools. In dogs and cats, the SI of hind limb PFz has been used to detect mild hind limb lameness when walking. In our study, the symmetry indices were similar to those reported in dogs [26]. These data support our clinical observations that the cats were not lame. We also recognized that minor radiographic changes, as are seen frequently in cats [27, 28], may not end in measurable lameness. Nevertheless, a report in dogs found that asymmetric joint moments can occur during trotting [29]. Consequently, more data from different breeds of lame and normal cats are needed to establish a reference range and to compare objective and subjective lameness analyses.

A difference in SI PFz (%) between fore- and hind limbs and an influence by breed might be explained by an hypothesis that forelimbs require more stability to manage the energy generated in the hind limbs, and that this effect seems to be larger in DSH cats.

In future, more studies are needed to evaluate kinematics and/or electromyogram (EMG) to gain more information on possible connections between breed variables and orthopaedic diseases. One additional advantage would be not only to evaluate vertical but also horizontal forces, as this could shed additional light on the biomechanics of the different breeds.

## Conclusion

In this study, we report the first ground reaction force data and temporospatial parameters in a group of purebred client-owned MC cats and compared them to DSH cats. We detected no differences in GRF measurements when normalised for %BM and %TF. Differences in temporospatial parameters, such as step length and paw contact area, were expected due to the larger body size of MC. MCs also displayed a shorter time to PFz in the hind limbs, and the reasons for this are unexplained by the reported data; thus, further investigations are needed.

## Supporting information

S1 File Raw data of all measured Domestic Shorthair and Maine Coon Cats. MC = Maine Coon, DSH = Domestic Shorthair, m = male, mc = male castrated, f = female, fc = female castrated, FL = fore left, FR = fore right, HL = hind left, HR = hind right, V = Velocity, PFz = Peak vertical force, Ifz = Vertical impulse, BM = Body mass, TF = Total force, SI = Symmetry index, SL = Step length, PCA = Paw contact area, SD = stance duration, StPh = Stance Phase.
(PDF)
