# Supplementary material for: Comparison of ground reaction force measurements in a population of Domestic Shorthair and Maine Coon cats
Source: PLoS One. 2018 Dec 12;13(12):e0208085. doi: 10.1371/journal.pone.0208085 (PMC6291092; doi:10.1371/journal.pone.0208085)
Supplement: S1 File — MC = Maine Coon, DSH = Domestic Shorthair, m = male, mc = male castrated, f = female, fc = female castrated, FL = fore left, FR = fore right, HL = hind left, HR = hind right, V = Velocity, PFz = Peak vertical force, Ifz = Vertical impulse, BM = Body mass, TF = Total force, SI = Symmetry index, SL = Step length, PCA = Paw contact area, SD = stance duration, StPh = Stance Phase. (PDF) [file pone.0208085.s001.pdf]

MC 1

Number	Breed	Sex	Date of Birth	Date of Measu	month	years
1	MC	m	06.07.09	27.04.15	69	5,8
2	MC	m	13.05.07	27.04.15	95	7,9
3	MC	m	29.03.11	27.04.15	48	4,0
4	MC	m	28.07.14	27.04.15	8	0,7
5	MC	m	15.08.14	27.04.15	8	0,7
6	MC	m	24.10.14	28.04.15	6	0,5
7	MC	m	03.07.14	28.04.15	9	0,8
8	MC	mc	10.11.11	28.04.15	41	3,4
9	MC	mc	01.01.09	29.05.15	76	6,3
10	MC	mc	19.05.14	19.04.16	23	1,9
11	MC	f	31.05.14	19.04.16	22	1,8
12	MC	f	02.04.07	04.05.16	109	9,1
13	MC	f	02.04.11	20.05.16	61	5,1
14	MC	f	05.02.12	20.05.16	51	4,3
15	MC	fc	08.07.13	20.05.16	34	2,8
						3,7
**DSH 0**						2,7

	Breed	Sex	Date of Birth	Date of Measu	month	years
16	DSH	mc	05.05.10	12.06.14	49	4,1
17	DSH	mc	02.07.14	24.02.15	7	0,6
18	DSH	fc	04.04.14	24.02.15	10	0,8
19	DSH	wk	01.05.08	24.02.15	81	6,8
20	DSH	wk	01.09.07	22.04.14	79	6,6
21	DSH	wk	01.05.12	19.02.15	33	2,8
22	DSH	wk	01.01.00	21.11.13	166	13,8
23	DSH	wk	01.11.09	31.03.15	64	5,3
24	DSH	mc	01.07.10	28.05.14	46	3,8
25	DSH	mc	01.07.10	28.05.14	46	3,8
26	DSH	fc	01.04.12	26.11.14	31	2,6
27	DSH	mc	01.01.06	06.02.15	109	9,1
28	DSH	mc		24.02.15		
29	DSH	fc		18.07.14		
30	DSH	fc	29.11.10	21.10.14	46	3,8

**body weight**	**speed**
7,5	0,74
6,9	0,83
7,1	1,04
3,9	0,82
5,7	0,78
3,1	0,65
4,1	0,60
6,1	0,69
5,9	0,86
6,5	0,74
7,9	0,57
6,8	0,52
8,5	0,55
7,5	1,10
6,3	0,67

**body weight**	**speed**	measurement from the PLOS
6	0,71	3
4,1	0,69	3
4,3	0,64	3
3,8	0,8	3
6,1	0,77	1
4	0,63	1
6,6	0,72	1
4,4	0,73	2
4,5	0,81	1
6	0,68	1
3,9	0,58	1
4	0,83	3
6,2	0,66	3
6,6	0,77	3
4	0,52	3

Number	Number in publication	Breed	Weight scale	FL_V	FL_PFz
1	1	1	7,5	0,74	41,84
2	2	1	6,9	0,83	35,20
3	3	1	7,1	1,04	42,61
4	4	1	3,9	0,82	18,29
5	5	1	5,7	0,78	30,27
6	6	1	3,1	0,65	16,44
7	7	1	4,1	0,60	20,48
9	8	1	6,1	0,69	32,57
10	9	1	5,9	0,86	35,39
11	10	1	6,5	0,74	41,36
12	11	1	7,9	0,57	42,31
13	12	1	6,8	0,52	39,39
14	13	1	8,5	0,55	54,43
15	14	1	7,5	1,10	49,39
16	15	1	6,3	0,67	33,85
1	16	0	6	0,71	31,80
2	17	0	4,1	0,69	22,60
3	18	0	4,3	0,64	22,19
4	19	0	3,8	0,8	22,69
5	20	0	6,1	0,77	34,87
7	21	0	4	0,63	21,33
8	22	0	6,6	0,72	38,32
9	23	0	4,4	0,73	24,89
10	24	0	4,5	0,81	23,17
11	25	0	6	0,68	29,54
13	26	0	3,9	0,58	19,73
14	27	0	4	0,83	21,79
15	28	0	6,2	0,66	39,64
16	29	0	6,6	0,77	32,89
17	30	0	4	0,52	25,36

	BM weight scale	FL_V	FL_PFz
Mean_1	6,25	0,74	35,59
SD_1	1,53	0,17	10,89
Mean_0	4,97	0,70	27,39
SD_0	1,11	0,09	6,58

1 Maine Coon
0 DSH

FR_PFz1	HL_PFz_1	HR_PFz1	FL_IFz1	FR_IFz1	HL_Ifz_1	HR_IFz1
42,43	30,01	30,60	16,64	16,88	10,28	10,66
36,51	29,06	28,91	12,90	12,68	10,55	9,97
43,10	24,21	22,76	12,84	12,60	6,05	6,38
18,65	17,89	17,16	4,30	4,71	4,18	4,14
31,29	25,77	25,22	10,65	11,53	9,34	8,59
16,77	13,86	13,64	4,72	4,87	3,81	3,95
21,98	17,80	17,97	7,48	8,16	6,45	6,78
30,98	27,22	29,18	12,45	11,21	9,61	10,74
34,64	24,05	22,33	11,26	11,18	6,85	6,13
39,62	26,54	25,50	14,29	13,99	8,97	7,78
41,16	32,98	34,30	16,64	16,88	10,28	10,66
38,47	30,91	31,81	18,43	16,55	14,05	13,48
56,14	41,56	43,58	21,03	23,24	15,37	16,74
51,79	28,09	25,67	17,12	17,20	8,92	8,42
34,19	23,92	24,97	13,10	13,56	8,46	8,81
30,88	29,23	29,80	10,24	10,67	9,36	9,50
23,46	17,07	16,26	7,22	6,92	4,96	4,60
21,63	18,97	18,47	7,05	7,07	6,22	6,43
22,23	8,88	10,04	5,80	5,90	2,50	2,56
33,99	20,88	19,77	11,48	11,05	6,46	6,09
21,51	14,96	16,34	6,40	6,18	4,41	4,64
38,33	30,86	29,51	14,36	15,51	11,02	11,19
26,63	15,33	16,01	8,29	9,12	4,94	5,53
22,66	20,76	21,06	5,70	5,75	5,31	5,31
28,30	26,00	23,52	10,34	9,86	8,75	7,71
20,25	15,26	13,90	7,03	7,17	5,01	5,04
22,71	16,14	14,20	6,07	5,94	4,35	3,79
39,46	26,90	25,74	15,73	15,63	10,93	10,29
34,37	21,99	23,49	9,80	9,57	6,37	6,40
25,61	19,73	20,72	9,70	8,98	7,44	7,43

FR_PFz1	HL_PFz_1	HR_PFz1	FL_IFz1	FR_IFz1	HL_Ifz_1	HR_IFz1
35,85	26,26	26,24	12,92	13,02	8,88	8,88
11,06	6,75	7,43	4,80	4,88	3,19	3,39
27,47	20,20	19,92	9,02	9,02	6,54	6,43
6,43	6,03	5,74	3,08	3,20	2,50	2,42

FL_PFz1_BM	FR_PFz1_BM	HL_PFz_1_BM	HR_PFz1_BM	FL_IFz1_BM	FR_IFz1_BM	HL_Ifz_1_BM
56,87	57,67	40,79	41,59	22,61	22,95	13,98
52,00	53,93	42,92	42,71	19,06	18,73	15,59
61,18	61,88	34,76	32,68	18,43	18,09	8,69
47,82	48,74	46,76	44,86	11,25	12,30	10,94
54,13	55,95	46,08	45,11	19,04	20,62	16,71
54,06	55,14	45,59	44,85	15,53	16,01	12,53
50,93	54,66	44,26	44,67	18,60	20,29	16,03
54,43	51,77	45,49	48,76	20,80	18,73	16,06
61,14	59,84	41,55	38,58	19,45	19,32	11,83
64,87	62,14	41,63	40,00	22,42	21,94	14,07
54,60	53,11	42,55	44,26	21,47	21,78	13,27
59,04	57,67	46,34	47,69	27,63	24,81	21,07
65,28	67,32	49,85	52,27	25,22	27,87	18,44
67,13	70,39	38,17	34,88	23,27	23,38	12,12
54,77	55,31	38,71	40,40	21,20	21,94	13,69
54,03	52,47	49,66	50,64	17,40	18,13	15,90
56,19	58,33	42,45	40,42	17,96	17,19	12,34
52,62	51,28	44,96	43,79	16,71	16,76	14,75
60,86	59,64	23,82	26,94	15,55	15,82	6,71
58,26	56,80	34,90	33,04	19,19	18,47	10,80
54,35	54,82	38,14	41,65	16,31	15,76	11,24
59,19	59,20	47,67	45,58	22,17	23,95	17,02
57,65	61,70	35,53	37,08	19,22	21,13	11,44
52,49	51,33	47,04	47,72	12,92	13,03	12,03
50,19	48,08	44,17	39,96	17,57	16,75	14,87
51,57	52,93	39,89	36,33	18,37	18,75	13,11
55,53	57,88	41,14	36,20	15,47	15,14	11,07
65,17	64,87	44,23	42,32	25,87	25,70	17,97
50,80	53,08	33,97	36,27	15,14	14,77	9,84
64,62	65,26	50,28	52,79	24,73	22,87	18,97

FL_PFz1_BM	FR_PFz1_BM	HL_PFz_1_BM	HR_PFz1_BM	FL_IFz1_BM	FR_IFz1_BM	HL_Ifz_1_BM
57,22	57,70	43,03	42,89	20,40	20,58	14,33
5,68	5,80	3,92	5,12	3,91	3,72	3,10
56,23	56,51	41,19	40,72	18,30	18,28	13,20
4,71	5,10	7,08	6,82	3,56	3,63	3,34

HR_IFz1_BM	FL_PFz1_TF	FR_PFz1_TF	HL_PFz_1_TF	HR_PFz1_TF	FL_IFz1_TF	FR_IFz1_TF
14,49	28,88	29,29	20,71	21,12	30,55	31,00
14,72	27,15	28,15	22,41	22,29	27,98	27,50
9,16	32,12	32,48	18,25	17,15	33,89	33,28
10,81	25,41	25,90	24,85	23,84	24,83	27,16
15,37	26,89	27,80	22,90	22,41	26,54	28,74
12,99	27,08	27,62	22,83	22,47	27,21	28,06
16,85	26,18	28,10	22,75	22,97	25,91	28,28
17,95	27,15	25,83	22,69	24,33	28,29	25,47
10,59	30,40	29,76	20,66	19,18	31,79	31,58
12,20	31,09	29,78	19,95	19,17	31,74	31,06
13,75	28,07	27,30	21,87	22,75	30,55	31,00
20,21	28,02	27,37	21,99	22,63	29,48	26,48
20,08	27,81	28,68	21,24	22,27	27,53	30,43
11,44	31,88	33,43	18,13	16,57	33,15	33,29
14,26	28,95	29,24	20,46	21,35	29,82	30,87
16,14	26,13	25,37	24,01	24,49	25,75	26,83
11,43	28,47	29,55	21,51	20,48	30,48	29,18
15,23	27,31	26,62	23,34	22,73	26,33	26,41
6,86	35,54	34,82	13,91	15,73	34,61	35,20
10,18	31,84	31,04	19,07	18,06	32,72	31,50
11,82	28,76	29,01	20,18	22,04	29,59	28,58
17,29	27,97	27,97	22,52	21,54	27,56	29,78
12,81	30,03	32,14	18,51	19,32	29,75	32,72
12,03	26,43	25,85	23,69	24,03	25,82	26,05
13,10	27,51	26,36	24,22	21,91	28,20	26,89
13,18	28,53	29,29	22,07	20,10	28,97	29,57
9,65	29,11	30,35	21,57	18,98	30,14	29,50
16,92	30,09	29,95	20,42	19,54	29,92	29,73
9,88	29,17	30,49	19,51	20,83	30,50	29,76
18,92	27,74	28,01	21,58	22,66	28,93	26,76

HR_IFz1_BM	FL_PFz1_GK	FR_PFz1_GK	HL_PFz_1_GK	HR_PFz1_GK	FL_IFz1_GK	FR_IFz1_GK
14,32	28,47	28,71	21,45	21,37	29,28	29,61
3,34	2,06	2,10	1,81	2,32	2,68	2,41
13,03	28,98	29,12	21,07	20,83	29,29	29,23
3,33	2,33	2,55	2,67	2,32	2,42	2,53

HL_Ifz_1_TF	HR_IFz1_TF	SI1_PFz_FL_1	SI1_PFz_HL_1	SI1_Ifz_FL_1	SI1_Ifz_HL_1	FL_SL_1
18,88	19,57	0,69	0,98	0,73	1,80	0,60
22,90	21,62	1,82	0,25	0,87	2,87	0,41
15,98	16,85	0,56	3,09	0,92	2,66	0,65
24,14	23,87	0,96	2,08	4,48	0,56	0,49
23,29	21,42	1,66	1,07	3,97	4,18	0,60
21,96	22,77	0,99	0,81	1,53	1,81	0,47
22,34	23,47	3,53	0,46	4,37	2,48	0,53
21,84	24,40	2,51	3,47	5,24	5,53	0,55
19,33	17,30	1,07	3,71	0,33	5,53	0,56
19,92	17,28	2,15	2,00	1,08	7,11	0,49
18,88	19,57	1,38	1,97	0,73	1,80	0,58
22,48	21,57	1,17	1,44	5,37	2,07	0,50
20,13	21,92	1,54	2,37	5,00	4,26	0,61
17,26	16,29	2,37	4,50	0,22	2,90	0,68
19,25	20,06	0,50	2,13	1,72	2,05	0,64
23,53	23,89	1,46	0,98	2,05	0,76	0,49
20,94	19,40	1,87	2,45	2,18	3,82	0,48
23,24	24,01	1,29	1,32	0,15	1,62	0,51
14,93	15,26	1,02	6,16	0,85	1,12	0,51
18,42	17,36	1,28	2,73	1,91	2,94	0,51
20,38	21,44	0,43	4,40	1,73	2,54	0,44
21,16	21,49	0,01	2,23	3,86	0,77	0,53
17,71	19,82	3,39	2,15	4,76	5,65	0,51
24,06	24,06	1,12	0,72	0,44	0,01	0,46
23,87	21,03	2,15	5,01	2,38	6,32	0,53
20,67	20,79	1,31	4,67	1,04	0,28	0,46
21,57	18,79	2,07	6,38	1,07	6,89	0,54
20,79	19,57	0,23	2,21	0,32	3,03	0,56
19,83	19,91	2,20	3,28	1,22	0,20	0,47
22,18	22,13	0,49	2,44	3,90	0,11	0,41

HL_Ifz_1_GK	HR_IFz1_GK	SI1_PFz_FL_1	SI1_PFz_HE_1	SI1_Ifz_FL_1	SI1_Ifz_HE_1	FL_SL_1
20,57	20,53	1,53	2,02	2,44	3,17	0,56
2,35	2,66	0,84	1,25	2,01	1,79	0,08
20,88	20,60	1,35	3,14	1,86	2,40	0,49
2,49	2,45	0,89	1,79	1,39	2,34	0,04

FR_SL_1	HL_SL_1	HR_SL_1	FL_PCA1	FR_PCA1	HL_PCA1	HR_PCA1
0,67	0,67	0,65	17,63	17,52	16,87	16,41
0,47	0,49	0,48	15,52	15,15	15,60	15,55
0,70	0,70	0,69	17,75	17,75	15,74	14,20
0,51	0,50	0,51	11,99	12,15	12,37	12,74
0,60	0,59	0,58	15,23	15,38	15,56	15,43
0,47	0,49	0,47	10,96	10,94	10,35	10,33
0,53	0,55	0,55	11,84	12,22	12,72	12,83
0,56	0,55	0,56	14,64	13,53	14,97	15,02
0,57	0,57	0,55	14,03	13,72	13,88	13,32
0,51	0,51	0,53	15,87	16,73	15,78	16,30
0,57	0,55	0,57	15,22	15,91	14,99	16,14
0,51	0,50	0,49	16,14	15,33	16,95	16,28
0,62	0,68	0,68	18,29	20,09	20,38	18,91
0,65	0,67	0,67	18,08	18,56	16,14	15,49
0,61	0,53	0,66	15,03	15,56	14,87	14,53
0,48	0,48	0,50	13,67	13,77	15,91	16,37
0,52	0,52	0,47	11,41	12,16	11,42	10,72
0,50	0,50	0,51	11,94	11,30	10,81	11,30
0,50	0,61	0,60	11,30	11,30	8,53	9,33
0,51	0,55	0,53	13,88	13,45	12,31	12,51
0,44	0,43	0,43	11,17	11,31	10,76	10,44
0,53	0,57	0,57	15,48	14,35	15,83	15,21
0,48	0,42	0,49	12,11	12,91	10,49	10,49
0,46	0,47	0,47	12,37	12,46	11,75	12,27
0,55	0,55	0,59	13,56	13,11	16,14	14,76
0,47	0,47	0,47	10,40	10,42	10,86	10,22
0,54	0,55	0,56	11,94	11,07	11,30	9,36
0,56	0,56	0,56	15,78	15,24	14,35	15,06
0,48	0,49	0,49	14,26	13,45	12,37	13,56
0,41	0,43	0,43	12,51	12,47	11,66	12,51

FR_SL_1	HL_SL_1	HR_SL_1	FL_PCA1	FR_PCA1	HL_PCA1	HR_PCA1
0,57	0,57	0,58	15,22	15,37	15,15	14,90
0,07	0,08	0,08	2,28	2,55	2,30	2,03
0,50	0,51	0,51	12,79	12,58	12,30	12,27
0,04	0,06	0,05	1,59	1,35	2,25	2,27

FL_SD1	FR_SD1	HL_SD1	HR_SD1	FL_StPh	FR_StPh	HL _StPh
0,51	0,52	0,50	0,48	54,37	52,70	30,04
0,54	0,51	0,49	0,47	62,48	67,17	42,40
0,40	0,38	0,34	0,38	51,24	39,79	31,39
0,33	0,35	0,33	0,34	68,31	63,37	36,10
0,51	0,53	0,50	0,47	64,23	70,12	32,91
0,42	0,42	0,38	0,40	63,12	67,60	43,21
0,53	0,53	0,50	0,52	60,82	58,65	39,50
0,52	0,49	0,48	0,51	56,06	60,34	42,26
0,45	0,45	0,42	0,40	65,76	65,50	35,10
0,50	0,51	0,48	0,43	58,59	55,20	49,31
0,69	0,75	0,76	0,70	50,21	53,03	45,39
0,67	0,62	0,64	0,61	57,25	52,88	48,83
0,54	0,58	0,51	0,54	66,67	57,50	34,24
0,53	0,50	0,46	0,50	47,37	55,18	33,91
0,52	0,52	0,49	0,48	44,17	53,16	36,03
0,43	0,46	0,42	0,42	55,43	45,99	35,14
0,41	0,40	0,38	0,37	56,72	50,84	34,96
0,46	0,47	0,44	0,45	63,99	67,23	60,55
0,38	0,40	0,40	0,37	61,03	55,88	51,84
0,45	0,45	0,41	0,42	60,00	67,24	59,09
0,43	0,41	0,38	0,39	56,58	61,31	39,06
0,54	0,57	0,51	0,54	63,53	61,23	39,40
0,48	0,50	0,45	0,46	63,99	64,76	60,94
0,34	0,35	0,35	0,35	48,25	48,65	38,81
0,46	0,47	0,46	0,43	50,36	46,69	35,25
0,48	0,47	0,44	0,47	58,97	60,60	47,31
0,37	0,35	0,34	0,33	55,19	54,88	38,24
0,56	0,56	0,53	0,51	68,59	62,15	39,53
0,43	0,42	0,39	0,38	64,74	64,34	45,65
0,53	0,51	0,51	0,50	54,60	63,67	51,75

FL_SPD1	FR_SPD1	HL_SPD1	HR_SPD1	FL_StPh	FR_StPh	HL _StPh
0,51	0,51	0,48	0,48	58,04	58,15	38,71
0,09	0,10	0,11	0,09	7,38	7,86	6,20
0,45	0,45	0,43	0,43	58,80	58,36	45,17
0,06	0,07	0,06	0,06	5,65	7,35	9,51

HR_StPh
32,01
46,39
43,68
38,94
38,81
41,13
38,60
40,73
40,91
38,00
60,91
44,43
34,93
44,80
40,39
42,71
52,61
50,25
49,73
65,77
48,02
41,11
67,77
27,76
37,86
40,08
53,29
51,62
38,79
51,25

HR_StPh
41,64
6,52
47,91
10,43
